# The methylation-independent mismatch repair machinery in *Pseudomonas aeruginosa*

**DOI:** 10.1099/mic.0.001120

**Published:** 2021-12-09

**Authors:** Yue Yuan On, Martin Welch

**Affiliations:** ^1^​ Department of Biochemistry, Hopkins Building, Tennis Court Road, Downing Site, University of Cambridge, Cambridge, CB2 1QW, UK

**Keywords:** hypermutation, MutS, MutL, mismatch repair, *Pseudomonas aeruginosa*

## Abstract

Over the last 70 years, we’ve all gotten used to an *Escherichia coli *-centric view of the microbial world. However, genomics, as well as the development of improved tools for genetic manipulation in other species, is showing us that other bugs do things differently, and that we cannot simply extrapolate from *E. coli* to everything else. A particularly good example of this is encountered when considering the mechanism(s) involved in DNA mismatch repair by the opportunistic human pathogen, *Pseudomonas aeruginosa* (PA). This is a particularly relevant phenotype to examine in PA, since defects in the mismatch repair (MMR) machinery often give rise to the property of hypermutability. This, in turn, is linked with the vertical acquisition of important pathoadaptive traits in the organism, such as antimicrobial resistance. But it turns out that PA lacks some key genes associated with MMR in *E. coli *, and a closer inspection of what is known (or can be inferred) about the MMR enzymology reveals profound differences compared with other, well-characterized organisms. Here, we review these differences and comment on their biological implications.

## Introduction

### Mutation is unavoidable

Evolution is underpinned, in part, by genetic variation. This, in turn, provides a pool of diversity in the population upon which natural selection can act. This genetic variation is driven by mutation of DNA. Although DNA is a very stable molecule – it is, after all, the hereditary material of most organisms – it is not immutable. For example, the DNA replisome is not *quite* perfect, and does sometimes introduce errors through straightforward enzymatic ‘slop’ (estimated at a rate of 10^−7^ bp^−1^ generation^−1^ [1]). Moreover, even in a perfect world, biology comes up against the thorny issue of chemistry; at the pH inside the cell, amino ↔ imino and keto ↔ enol tautomerism are a fact of life, and indeed, are essential for some of the most important reactions in biology (e.g., the pyruvate kinase reaction [2]). This tautomerism can lead to some unusual base pairing in DNA. For example, if, at the instant of incorporation into a nascent DNA strand, the cytosine side chain on an incoming dCTP happens to adopt the *imino* configuration in place of the normal amino configuration, a C_imino_-A_amino_ base pair is possible. Similarly, if the thymine side chain on a template strand happens to adopt the *enol* configuration, a T_enol_-G_keto_ pairing is possible. Such inappropriate tautomeric pairings are stable (once the hydrogen bonds have formed) but give rise to subtle deformations of the B-DNA helical structure. Although this ‘rare tautomer’ hypothesis has been in circulation for many years now, there is now direct crystallographic evidence in support of it as a major driver behind the more common ‘transition’ mutations (leading to purine→purine and pyrimidine→pyrimidine substitutions) [3, 4]. Finally, base ‘wobble’ can also lead to incorrect base incorporation and therefore, additional mismatches.

If not identified and repaired, mismatches can become replicated and thence, lead to mutation (although strictly speaking, a mutation is a polymorphism that becomes ‘fixed’ through selection in the population). Most such mismatches are corrected immediately by the 3′→5′ exonuclease (‘proofreading’) activity of the replisome. However, some mismatches inevitably escape detection. Fortunately though, there is a post-replicative surveillance system (although in some organisms, this is probably best described as a co-replicative mechanism) at hand that constantly carries out ‘quality control’ of newly-synthesised DNA; the ‘mismatch repair’ (MMR) machinery. Although the MMR system is best-known for detecting mismatched bases, it can also recognise and repair short (1–4 bp) indel loops e.g. arising from replicative slippage. In the absence of a functional MMR system, the mutation rate of cells increases by around 10^3^-fold [5–7].

### The basic principles of MMR

MMR involves four basic steps; (i) scanning and detection of mismatches on DNA, (ii) discrimination between parental and daughter strands, (iii) nicking of the daughter strand and removal of the error-containing nascent strand, and (iv) re-synthesis of the new daughter strand [8–11]. The daughter-strand specificity of the MMR means that any damage or lesions on the parental strand are not repaired by the MMR machinery [12].

### The *E. coli* paradigm

The MMR machinery in *Escherichia coli* (Ec) has been well characterised compared with other bacteria (although the jury is still out regarding key mechanistic details). In essence, by 1989, *in vitro* reconstitution experiments with purified components had revealed that efficient MMR requires only a mismatch recognition protein (MutS_Ec_), an accessory protein (MutL_Ec_), an endonuclease (MutH_Ec_), a helicase (UvrD_Ec_) and one of four redundant exonucleases (RecJ; a 5′→3′ exonuclease, ExoVII; a bidirectional exonuclease, and ExoX/ExoI; 3′→5′ exonucleases) [13–15]. Single strand-binding protein (SSB) plays a non-essential role by protecting transiently exposed single-stranded regions, and gap re-synthesis/nick repair is carried out by the usual DNA replicative machinery of the cell [16].

In *E. coli *, DNA is methylated by Dam methylase on the N^6^ position of the adenine base in the sequence dGATC [17, 18]. However, this is a relatively slow process, so immediately after a new daughter strand is synthesised, the DNA duplex is essentially hemi-methylated, with methylation on the parental strand only. This hemi-methylation allows the MMR machinery to effectively discriminate between the methylated parental strand and the non-methylated nascent daughter strand. Shortly after replication, MutS_Ec_ dimers assemble on the DNA and execute a bidirectional 2D scan. MutS_Ec_ is also known to associate with the replisome β-sliding clamp, which doubtless facilitates this scanning mechanism [19, 20]. Upon the detection of a mismatched base pair, MutS_Ec_ changes its conformation and recruits a coupling protein, MutL_Ec_, in an ATP-dependent reaction. This recruitment of MutL_Ec_ is the trigger for downstream activation of the remainder of the MMR cascade. The MutS_Ec_-MutL_Ec_ complex activates the endonuclease activity of MutH_Ec_, which generates a single-stranded nick (either 5′ or 3′ of the mismatch) on the unmethylated strand of DNA at the nearest hemi-methylated dGATC site [16]. This mechanism requires ‘action at a distance’, probably via looping of the DNA, thereby allowing the MutS_Ec_-MutL_Ec_ complex to physically contact MutH_Ec_ at a distant (up to 1 kb away) hemi-methylated dGATC site. The MutS_Ec_-MutL_Ec_ complex then directs the helicase, UvrD_Ec_ to the nick, allowing unwinding of the helix. Curiously, this loading of UvrD_Ec_ is directional, such that the duplex is unwound from the nick only in the direction of the mismatch. Concomitantly, one of the four exonucleases mentioned above follows the helicase, digesting the daughter strand in the direction of the mismatch. This exonucleolytic activity continues to a position just beyond the mismatch. Concomitant with the exonuclease action, SSB coats the exposed parental strand [16, 19]. Finally, the digested strand is replaced through PolIII holoenzyme-directed re-synthesis, and the resulting nick is sealed by ligase to restore full continuity of the DNA [21].

### *Pseudomonas aeruginosa* does things differently

It is worth reiterating that Dam-dependent strand discrimination and MutH endonuclease are central to the *E. coli* MMR machinery. However, the vast majority of bacterial species lack Dam methylase and MutH (an overview of which proteins are present/absent in a selection of prokaryotes is shown in Table 1) [9, 19, 22–26]. Indeed, it has been postulated that MutH is a relatively recent evolutionary development [27], and as noted by Putnam, only a few gammaproteobacteria possess methyl-directed MMR systems [24]. This is particularly relevant in the case of the opportunistic human pathogen, *P. aeruginosa* (PA). This organism is often associated with human soft tissue infections, and especially chronic infections in the airways. Perhaps the best characterised infection scenario is in people with the inherited genetic disorder, cystic fibrosis (CF). For reasons that are still not entirely clear, people with CF appear to be exquisitely predisposed to PA infection [28, 29]. These CF-associated infections are treated with aggressive antibiotic interventions, yet resistance to these agents eventually becomes all but inevitable [30]. This resistance is often accompanied by the isolation of variants from the airways with loss-of-function mutations in *mutS* [31, 32]. The reason for this linkage between antimicrobial resistance (AMR) and *mutS* mutation is that loss of *mutS* function leads to hypermutability. Indeed, PA *mutS* mutants typically mutate at a rate 10^2^–10^3^ times faster than the wild-type and display heterogeneity with respect to phenotype [33, 34]. This, in turn, increases the frequency with which loss-of-function mutations arise in repressors of e.g. multi-drug efflux pumps, leading to inappropriate expression of these pumps and consequent elevated resistance to antimicrobial agents. For example, loss of function mutations in *nfxB*, a repressor of the *mexCD-oprJ* multidrug efflux pump, elicits a sudden step increase in the resistance of the organism against a range of fluoroquinolones, macrolides and certain β-lactams [35–37]. This notwithstanding, and somewhat surprisingly (given its clinical importance) little is currently known about MMR in PA. In the remainder of this review, we assess the current state-of-the-art with regards to MMR in this organism.

**Table 1.** Distribution of key MMR proteins (and HsdM) in different bacterial species (✓ indicates the number of paralogues of each gene present)

Species	Dam	HsdM*	MutS	MutL	MutH
* Bacillus subtilis* 168			✓✓	✓	
* Aeromonas hydrophila* ATCC7966	✓		✓	✓	✓
* Pectobacterium atrosepticum* SCRI1043	✓	✓	✓	✓✓	✓
*Escherchia coli* MG1655	✓	✓	✓	✓	✓
* Haemophilus influenzae* 10810	✓	✓	✓	✓	✓
* Klebsiella pneumoniae* KPNIH24	✓✓		✓	✓	✓
* Proteus mirabilis* CYPV1	✓		✓	✓	✓
* Vibrio cholerae* O1 El Tor N16961	✓	✓	✓	✓	✓
* Yersinia pestis* CO92	✓		✓	✓	✓
* Yersinia pseudotuberculosis* YPIII	✓✓✓	✓	✓	✓	✓
* Campylobacter jejuni* ATCC 700819†		✓	✓‡		
* Francisella tularensis* sp. novicida* U112*		✓✓	✓	✓	
* Helicobacter pylori* 266955†		✓✓✓	✓‡		
*Acinetobacter baumanii* ATCC17978			✓✓	✓✓	
* Hahella chejuensis *		✓✓	✓	✓	
* Pseudomonas aeruginosa *		✓	✓	✓	
* Ralstonia solanacearum* GMI1000		✓	✓	✓	
* Neisseria meningitidis* MC58	✓§	✓	✓	✓	
* Burkholderia cepacia* ATCC25416			✓	✓	
* Azotobacter vinelandii* DJ		✓	✓	✓	
* Sinorhizobium meliloti* 1021		✓	✓	✓	
* Xanthomonas campestris* B100		✓✓	✓	✓	
* Stenotrophomonas maltophilia* K279a			✓	✓	
* Geobacillus stearothermophilus *		✓‡✓✓✓	✓✓	✓	
* Mycoplasma pneumoniae* M129†		✓			
* Rhodospirillum rubrum* ATCC1170			✓	✓	
* Staphylococcus aureus* RF122		✓✓	✓✓✓	||	
*Borellia burgdorferi* B31		✓‡	✓✓	✓	
* Chlamydia trachomatis* D/UW-3/CX			✓	✓	
*Bacillus anthracis Ames*			✓✓✓	✓	
* Corynebacterium glutamicum* ATCC13032†					
* Mycobacterium tuberculosis* H37Rv†		✓			
* Clostridium difficile* 630		✓‡	✓✓✓✓	✓	
* Listeria monocytogenes* EGD-e	✓‡		✓✓	✓	
* Streptococcus pneumoniae* TIGR4		✓✓	✓	✓	
*Treponema pallidum (Nichols*)	✓		✓	✓	
* Thermotoga maritima* MSB8			✓✓	✓	
*Nitrosomonas eutrophia*		✓	✓✓	✓	
* Rickettsia rickettsii* Colombia			✓	✓	
*Bordetella pertussis Tohama I*			✓	✓	

*Not part of MMR mechanism for methylation (to be discussed in section below).

†These species do not possess most of the MMR protein homologues suggesting that they may use a different system for DNA repair.

‡Relatively high E-values indicating low similarity between the homologs.

§In truncated form.

||Though absent in *Staphylococcus aureus* RF122, a MutL homolog is present in strain MSSA476.

### *P. aeruginosa* lacks Dam methylation

Although PA does encode an adenine methyltransferase activity (mediated by the *hsdMSR* genes), there is no direct evidence implicating DNA methylation in MMR. Only 0.1% of all adenine bases are methylated in PA strain PAO1, and this declines to an undetectable level in an *hsdMSR* mutant. Intriguingly, the HsdM methyltransferase appears to have a hitherto unexpected function in the epigenetic regulation of pathogenicity [38]. We further note that the *hsdMSR* cluster is encoded on the accessory genome, which would not be expected for a crucially-important (and presumably, highly-conserved) function such as MMR [38, 39]. Furthermore, and in comparison with other species, it is obvious that there is no correlation between the presence/absence of HsdM and any other MMR enzymes, especially Dam and MutH (Table 1). Therefore, the question of how PA discriminates between parental and daughter strands is not clear. One possibility is that, like the human and *Saccharomyces cerevisiae* MMR systems [40, 41], PA determines strand specificity via monitoring of the natural discontinuities associated with normal DNA replication, such as the termini of Okazaki fragments. Consistent with this, there are indications that MMR is indeed more efficient on the lagging strand rather than the leading strand [42]. However, the mechanism by which mismatches in the nascent leading strand might be authorized for repair remains unclear [40].

### *P. aeruginosa* lacks a MutH homologue

Similar to many other organisms, PA lacks any obvious homologue of the *E. coli* endonuclease, MutH [25, 26, 43]. This has led to the suggestion that MMR is initiated at naturally-occurring nicks in the DNA. In addition to the termini of Okazaki fragments, nicks may arise from the degradation of mis-incorporated ribonucleotides by RNase HII, or due to oxidative damage [44–46]. If this is correct, the enzymatic ‘slop’ leading to mis-incorporation of ribonucleotides into DNA may be evolutionarily important, since without it, nicks would be rarer and MMR efficiency would decline. However, it has recently been shown that MutL can directly generate the nick as strand discrimination signal following activation by the β-clamp component of the replication machinery. This mechanism has been inferred in eukaryotes and *B. subtilis* [47–49] and there is considerable accruing evidence to suggest that PA employs a similar mechanism (see section on MutL, below).

### MutS_PA_

As in other organisms, it appears that MutS plays the central role in *P. aeruginosa* MMR. *MutS*_PA_ expression is negatively controlled by the stationary phase sigma factor, RpoS. An obvious corollary of this is that mismatch repair is depressed in the stationary phase (as is DNA synthesis, so this makes sense) [50, 51]. The activity of MutS_PA_ is also temperature-sensitive; the DNA-binding capability and ATPase activity of MutS_PA_ are inhibited when the temperature exceeds 20 °C for more than a few minutes [52]. Whether this temperature sensitivity has any physiological significance is not clear; although it is tempting to speculate that growth in a human host may accelerate the mutation rate through decreased MutS activity, there is little evidence to support this notion.

What is clear though, is that MutS_PA_ is likely more conformationally dynamic than MutS_Ec_. Unlike its enteric cousin, the *P. aeruginosa* protein can adopt dimeric and tetrameric configurations, and readily aggregates to form higher-order structures too. Furthermore, and in contrast with MutS_Ec_, where the dimer has been shown to be sufficient for DNA repair [53], MutS_PA_ binds more tightly to DNA as a tetramer [52, 54, 55]. The formation of MutS_PA_ tetramers is strongly-dependent on residues in its C-terminal region (in particular, R842 and K852). Deletion of these residues leads to failure to oligomerise and loss of function [54]. Extending this ‘compare and contrast’ analogy, although MutS_PA_ (855 amino acids) shares a high degree (59%) of identity with MutS_Ec_ (853 amino acids), MutS_PA_ is dominant-negative when introduced into *E. coli *, and moreover, fails to complement a *mutS*-deficient *E. coli* strain [56]. It is not yet clear why this is, although one obvious possibility is that key protein-protein interactions may be abolished due to these variations in MutS primary sequence.

In canonical MMR systems such as those in *E. coli* and *B. subtilis *, MutS is thought to interact with the β-sliding clamp of the replisome complex, thereby facilitating scanning, and tightly coupling (both spatially and temporally) MMR with replication [20, 57–60]. MutS does this because it contains a conserved ‘clamp binding motif’ (CBM) which binds tightly to a discrete pocket on the β-clamp [57]. It has been suggested that this association of MutS_PA_ with β_PA_ may impede interaction of MutS_PA_ with MutL_PA_ [55]. However, in *E. coli *, recognition of a mismatched base pair by MutS_Ec_ leads to its detachment from β_Ec_, thereby allowing recruitment of MutL_Ec_ [61], and presumably, the same mechanism is employed in *P. aeruginosa *. Mismatch recognition in *E. coli* also involves the so-called mismatch recognition motif (MRM; Phe-Xaa-Glu), and this motif is conserved in MutS_PA_. It used to be thought that MutS binding to mismatches in DNA leads to kinking of the duplex, and that this might be a/the signal stimulating downstream events. However, more recent work has shown that the conserved phenylalanine residue in the MRM plays an important role by intercalating between the bases at the mismatch site and that this actually leads to straightening of the DNA [62]. Curiously, MutS_PA_ contains two consecutive identical MRMs (F_33_YELFYE_39_). Once detected, mispaired bases are thought to form a hydrogen bond with the conserved Glu residue in the MutS_Ec_ MRM, and the same likely also applies to MutS_PA_ [63–66]. Concomitantly, ATP is hydrolysed to ADP, driving a conformational change in MutS_PA_ that increases its affinity for the heteroduplex [19, 52, 67–70]. However, and in spite of the assistance it provides in orientating and localising MutS, β_PA_ cannot itself be considered as a component of the MMR machinery because the mutation rate of a *P. aeruginosa mutS* ^β^ mutant (in which the clamp binding motif is incapable of binding β_PA_) is comparable to that of the wild-type [60].

MutS_PA_ also has ‘non-classical’ MMR-associated functions. For example, in the presence of reactive oxygen species (ROS), *mutS* displays epistasis with the *dinB*-encoded error-prone polymerase, Pol IV [71]. During Pol III-mediated DNA replication, Pol IV is recruited to β_PA_ where it plays an important role in removing stalled replication forks [72–76]. However, and being error-prone, Pol IV sometimes introduces mismatches. When it does so, MutS_PA_ displaces Pol IV by binding to β_PA_ via its clamp-binding motif, thereby initiating the required repair [55, 58, 60, 77]. One obvious question is why should the cell employ an error-prone polymerase (Pol IV) under *any* circumstances? An anthropocentric response is that under the genotoxic conditions in which Pol IV is induced, a little bit of infidelity might not be a bad thing; although it may accelerate the mutation rate, the accompanying increased genetic diversity may offer some *‘in extremis’* last-ditch evolutionary solutions that enhance survivability in these circumstances. Well, that’s the theory, anyway, and at least the cell does its best to repair these errors.

### MutL_PA_

Apart from MutS, MutL is the other conserved core and multifunctional MMR protein in PA. Unlike *B. subtilis* and *Listeria monocytogenes *, in which *mutS* and *mutL* are encoded together in a discrete gene cluster, in PA, *mutS*_PA_ (PA3620), *mutL*_PA_ (PA4946) and *uvrD*_PA_ (PA5443) are dispersed across the PA genome. As noted earlier, MutL_PA_ likely contributes to the initial strand cleavage event during nascent strand discrimination, and also towards mediating base excision and UvrD recruitment in the later stages of the MMR mechanism. A recent study of CF-derived PA hypermutators revealed a greater number of mutations in *mutL* than in *mutS*, which is unexpected given the smaller size of the *mutL* gene (1902 bp) compared with *mutS* (2568 bp) [35]. However, it may simply be that the *mutL* ORF contains more repetitive DNA (‘homopolymeric tracts’) giving rise to slippage, although this is not immediately obvious from inspection of the sequence. Just as in *E. coli *, in the presence of ATP, mismatch-bound MutS_PA_ recruits MutL_PA_ and forms a transient ternary complex [78–82]. However, and because PA does not encode any obvious endonuclease (*mutH*) homolog, it seems that MutL_PA_ itself is likely responsible for creating the required nick, as is the case in many other MutH-less organisms [83, 84]. The MutL_PA_ endonuclease can cleave both single- and double-stranded DNA [83–85]. MutL is a ‘GHKL-type ATPase’ (**G** yrB, **H** sp90, Histidine **K** inase and Mut **L**), with the ATPase activity associated with its conserved N-terminal domain (NTD) [56, 86–89]. The ATPase activity in the MutL_PA_ NTD regulates the endonuclease activity of the protein, which is associated with the adjacent C-terminal domain (CTD) [90]. Clearly, the MutL in organisms that encode a *mutH* endonuclease homolog lack this CTD. The endonuclease activity in the CTD is activated upon binding of Mg^2+^ or Mn^2+^ (but is inhibited by Zn^2+^) [65, 83, 85, 90] and is associated with a motif (D(Q/M)HA(X)_2_E(X)_4_E) that is conserved in the CTD of other MutH-less species such as *Aquifex aeolicus *, *Thermus thermophilus* and *Neisseria gonorrhoeae* [65, 83, 91–93]. Interestingly, and by contrast, Zn^2+^ is essential for *B. subtilis* MutL endonuclease activity *in vivo* [49].

MutL_PA_ has a preference towards nicking terminal-less circular DNA rather than linear DNA in physiological conditions [84]. However, and whereas MutH_Ec_ only cleaves at dG-^Me^A-TC sites, MutL_PA_ generates non-specific nicks anywhere. In the worst-case scenario, it can even cause double stranded breaks (DSB), which could be catastrophic for the cell. This can happen even in the absence of a mismatch in the DNA, and is independent of MutS_PA_, similar to some other bacterial MutL proteins and eukaryotic MutLα [47, 49, 83–85, 91, 92, 94, 95]. If DSB do arise, these are repaired by either the error-prone non-homologous DNA end-joining (NHEJ) machinery [96–98] or by the homologous recombination (HR) pathway [99–101]. The caveat of DSBs notwithstanding, the sequence-independent incision catalysed by MutL offers the advantage of being less-dependent on the distribution of GATC sequences than MutH-dependent incisions [102].

Notwithstanding the HR and NHEJ repair machinery, the problem still remains that MutL_PA_ endonuclease (‘nicking’) activity can be MutS_PA_-independent. Clearly, this could be potentially disastrous, if unchecked. However, this unrestrained endonuclease activity is suppressed in the presence of ATP (in spite of the fact that the MutL_PA_•ATP complex remains competent for loading onto DNA) [65, 83, 91, 92]. Moreover, the endonuclease activity of MutL_PA_ is enhanced upon interaction with MutS_PA_ [90]. When this happens, the ATPase activity in the NTD of MutL_PA_ enhances the endonuclease activity in the CTD of the protein, which, in turn, creates a single-stranded nick in the bound DNA [83, 84]. It is worth mentioning that in *E. coli *, the MutL•ATP complex also stabilizes attachment of MutS_Ec_ to the site of the DNA mismatch, and this is probably the same in *P. aeruginosa* too [19, 103, 104].

The MutL CTD in some organisms contains a negatively-charged patch, raising the question of how/why the protein exhibits affinity for DNA in the first place. However, in these organisms, DNA-binding may be facilitated via direct interaction of MutL with the β-sliding clamp of the replisome, which occludes the negatively charged patch [49]. For example, the MutL CTD from *B. subtilis* and *N. gonorrhoeae* contains a motif, QXX(L/I)XP, which binds the β-clamp, and it has been suggested that this may assist in correctly orienting the MutL at DNA mismatches [23, 49, 105]. However, this type of β-MutL interaction may not be universal. Fortunately though, Fukui *et al*. have divided MutL into three sub-families, with only sub-family I containing the β-binding motif and negatively-charged patch [105]. MutL_PA_ does indeed fall into this sub-family and contains a putative β-binding motif (Q_492_PLLVP_497_) in the CTD. Taken together, these data favour a model in which the endonuclease activity of MutL_PA_ is normally inhibited by ATP. After recruitment by MutS_PA_ at a mismatch (a delivery process that may involve the replisome β_PA_), the ATPase activity in the NTD of MutL_PA_ is triggered, leading to relief of the endonuclease inhibition (possibly mediated via altered domain-domain or protein-protein interactions) and subsequently, nicking of the nascent DNA strand by the MutL_PA_ CTD.

One particularly intriguing observation is that MutL_PA_ can complement an *E. coli* Δ*mutL* mutant. This is somewhat surprising, given that MutL_Ec_ and MutL_PA_ share only 18% amino acid sequence identity in the crucial endonuclease-encoding CTD. This said, most (21/22) of the key amino acid residues known to be important for MutL_Ec_ function are conserved in MutL_PA_, and the structural framework of the CTD also seems to be conserved between the two proteins [106, 107]. One possibility is that the MutH-independent nicks introduced by MutL_PA_ are sufficient for MMR in *E. coli *. However, the CTD of MutL_PA_ can also interact with MutH_Ec_ *in vitro*, suggesting direct restoration of a functional protein-protein interaction is possible. The evolutionary rationale for maintaining this interaction (or interacting surface) is not clear, and hints that there is potentially much more to discover here.

### UvrD_PA_

In the *E. coli* MMR system, the UvrD helicase activated by MutL_Ec_ functions to unwind the DNA. This unwinding (3′→5′, relative to the mismatch) is oriented by the MutS•MutL•UvrD•DNA complex in the presence of ATP [48, 108–110]. *P. aeruginosa* encodes a UvrD homologue, although biochemical confirmation of a MutL_PA_-UvrD_PA_ interaction has not yet been reported. In both *P. aeruginosa* and the related species, *Pseudomonas putida *, *uvrD* mutants display hypermutability, although not to the same extent as *mutS* and *mutL* mutants [111, 112]. This suggests that other helicases may be able to step in and carry out a similar function. Apart from its role in MMR, UvrD is also involved in the nucleotide excision repair (NER) mechanism induced by ultraviolet light [113]. However, the functions of UvrD_PA_ in MMR and NER are separable; mutations in the conserved ATP binding motif of UvrD_PA_ have a significant impact on MMR, but a much lower impact on NER [56]. Interestingly in this regard, and like MutS_PA_, UvrD_PA_ is unable to complement an *E. coli uvrD* mutant, despite the high sequence similarity of UvrD in both species [56, 113]. This makes the observation outlined above, that MutL_PA_ can complement an *E. coli mutL* mutant, all the more surprising, especially given the very low sequence conservation between those two proteins.

### Exonuclease

Upon unwinding the region around the error by UvrD helicase, a segment of the erroneous strand is digested by exonuclease. Little is currently known about which particular exonuclease is involved in MMR_PA_ and our current understanding is limited largely to gene presence/absence relative to *E. coli *. The *E. coli* exonucleases are RecJ, ExoVII, ExoX and ExoI. RecJ_PA_ (PA3725) is 58% identical to RecJ_Ec_. On the other hand, and whereas *E. coli* ExoVII is comprised of two subunits, namely XseA and XseB, PA does not possess the larger XseA subunit, and its smaller XseB subunit (PA4042) is only 50% identical to the *E. coli* orthologue. Furthermore, in PA, the *xseB* gene is operonic with a geranyltransferase encoded by *ispA*, and does not likely function as an exonuclease. ExoX is also absent in PA, although an ExoI/SbcB orthologue (PA4316) is present and shares 51% identity with *E. coli* ExoI. Like *E. coli *, PA also encodes single-stranded binding protein (SSB; PA4232) to protect the template from degradation during repair. PA4232 is 75% identical to SSB_Ec_.

### Concluding comments and unresolved questions

In addition to the abovementioned enzymes, there are a number of other anti-mutator proteins found in PA, although these all appear to be involved in very specific circumstances and cannot be considered to be components of the ‘core’ MMR machinery. For example, MutT, MutY and MutM are involved in the 7,8-dihydro-8-oxo-deoxyguanosine (8-oxo-dG or ‘GO’) repair system [114–116] and there is some evidence that PfpI may be involved in DNA repair following oxidative damage [117].

**Fig. 1.**
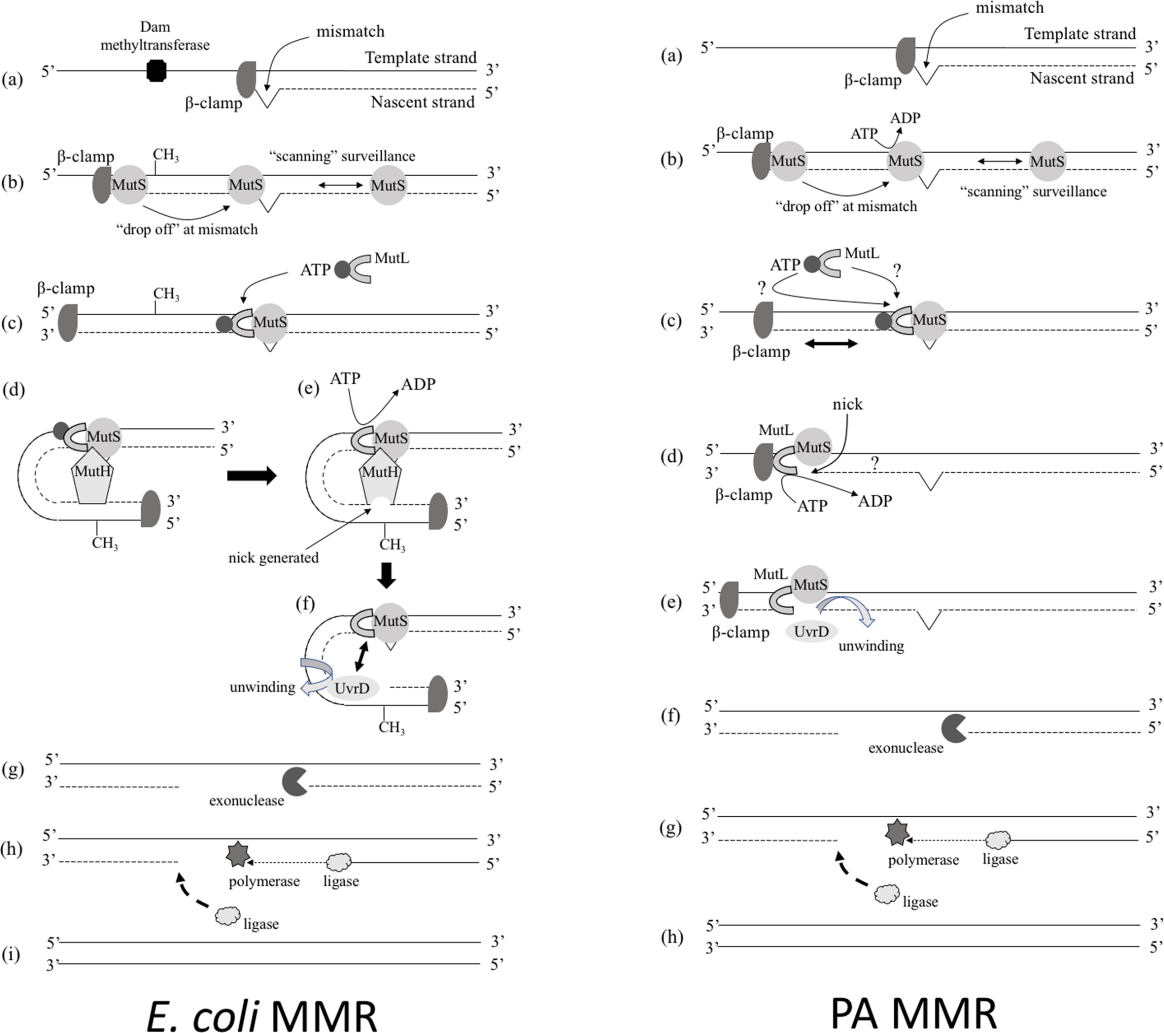
A comparison showing the canonical *E. coli* MMR pathway and a proposal for non-methyl-directed MMR in *P. aeruginosa *. In *E. coli* (left panel) the nascent strand is hypomethylated relative to the template strand (**a**). MMR begins with ATP-bound dimeric MutS binding to the site of a mismatch (**b**). This may be via carriage on the β subunit (‘sliding clamp’) of the replisome, or simply through continual bidirectional ‘scanning’ of the genome. Upon recognition of a mismatch, MutS changes its conformation and recruits MutL in an ATP-dependent reaction (**c**). This, in turn, subsequently leads to recruitment of MutH, which nicks the nascent DNA strand opposite a nearby Dam-methylated adenine base. The MutH endonuclease activity dependent on ATP hydrolysis by the MutS-MutL-MutH complex, and likely involves bending of the DNA if ‘action at a distance’ is required (**d, e**). Following nicking of the hypomethylated strand, the MutS-MutL complex recruits the UvrD helicase, which unwinds the duplex in the direction of the mismatch (**f**). The exposed single-stranded hypomethylated DNA, protected by SSB, is then digested by one of the exonucleases present, to a point beyond the original mismatch. The extent of retrograde (3′ → 5′) digestion is presumably limited by the processivity of the exonuclease (**g**). The resulting gap is then filled in and sealed through the combined action of DNA polymerase and DNA ligase (**h, i**). Some of the reactions in PA (right panel) are superficially similar, although many of the details are yet to be elucidated, so the presented model is inevitably a simplification. The key difference between PA and *E. coli* is that neither DNA strand is significantly methylated in the former (**a**). Current evidence suggests that MutS tetramers recognize DNA mismatches during DNA replication and are delivered to these sites via the β-sliding clamp, although post-replicative scanning surveillance also seems likely (**b**). DNA binding by MutS is accompanied by ATP hydrolysis (and concomitant release of the β-clamp) and is followed by recruitment of ATP-inhibited MutL. Again, this delivery of MutL to the MutS-bound mismatch may be via the β-clamp during replication, or via the β-clamp post-replicatively, or independent of the β-clamp (**c**). In the ATP-bound form, the CTD-associated endonuclease activity of MutL is inhibited. However, upon binding MutS, the ATPase activity of the NTD of MutL becomes enhanced, thereby relieving the ATP-dependent inhibition of the CTD-endonuclease. This leads to the generation of a nick (**d**). Quite how far away from the original mismatch this nick is, or whether the process involves DNA bending (as seems likely and as inferred for *E. coli* MMR) is not yet clear. Nor is it clear how strand discrimination is maintained. [We note that the model proposed here implies that MutS physically moves away from the mismatch as a complex with MutL before the nick is made. However, it is equally likely that MutS remains bound to the mismatch and that the interaction between dynamic β-bound MutL and ‘static’ MutS is achieved through DNA bending.] Following nicking of the DNA on the nascent strand, UvrD is recruited (**e**) and unwinds the DNA towards the mismatch. Again, how this directionality is ensured is not clear. Subsequent exonucleolytic degradation of the nascent strand and ‘fill-in/polishing’ are presumably the same as in *E. coli *.

Our current understanding of MMR in PA compared with MMR in *E. coli* is summarised in Fig. 1. Interestingly, and based on the known genetics and biochemistry, this mechanism is more similar to that proposed for a Gram-positive species, *B. subtilis *, than it is for *E. coli* (which, like PA, is a Gram-negative organism). A comparison of the MMR enzymology in each species is also shown in Table 2 (modified from Lanhert *et al*) [25, 26].

**Table 2.** Comparison between the MMR proteins in *E. coli *, *P. aeruginosa* and *B. subtilis
*

Protein	* E. coli *	* P. aeruginosa *	* B. subtilis *
Methylase	✓ (Dam methylase)	✓ (HsdM methylase; not used for MMR)	✕
β-clamp	✓	✓	✓
MutS	✓	✓	✓
MutL	✓ (but no endonuclease activity)	✓ (has endonuclease activity)	✓ (has endonuclease activity)
MutH	✓	✕	✕
Helicase	✓ (UvrD)	✓ (UvrD)	✓ PcrA (=UvrD) and YrrC

However, although likely correct in outline, some of the proposed steps remain unclear, and we have taken the liberty of identifying some of the associated unresolved questions below;

i. Does nascent strand discontinuity (e.g. Okazaki fragments) act as a signal for strand discrimination in *P. aeruginosa *, or does MutL play a far more active role? To what extent is MMR possible in the absence of active DNA replication? What determines the frequency of nicking by MutL? Does MutL nicking activity depend at all on the frequency of naturally-occurring nicks in the DNA?

ii. Does β_PA_ pause at the MutS_PA_-marked mismatch? Does β_PA_ function independent of the replisome to ‘scan’ the DNA during non-replicative periods? How does β_PA_ deliver MutL_PA_ to the MutS-marked mismatch? Does the MutS_PA_-MutL_PA_ complex subsequently move as a unit away from the mismatch before MutL-dependent nicking occurs?

iii. What regulates expression of the MMR machinery? Is there any physiological significance to the apparent thermosensitivity of MutS_PA_?

iv. Recent work has implicated redox-associated ‘sensors’ in DNA repair, especially proteins with [4Fe-4S] centres [118–126]. The model here (developed largely by the innovative discoveries made by Jacqueline Barton’s team) is that the stacked bases in DNA act as an ‘electron wire’, allowing charge transfer (CT) from lesions through the surrounding DNA [118]. This charge transfer and can be detected by DNA-bound [4Fe-4S]-containing proteins. In essence, CT through DNA to the redox protein is proposed to lead to dissociation of the latter from the DNA. Lesions (and possibly tautomeric mismatches too) affect the efficiency of this CT, leading to an accumulation of undissociated DNA-bound redox protein around the site of the lesion, thereby ‘marking it’ for repair. In this regard, although MutS_PA_ does not contain any iron-sulfur centres, the *mutS* gene in PA is encoded alongside a ferredoxin (*fdxA*) and is predicted to be operonic with this. This ‘redox’ chapter in the MMR story is still in its relatively early days, but we strongly recommend the reader to ‘watch this space’…

v. The ‘slop rate’ (inefficiency) of the MMR and DNA replicative enzymes seems to be evolutionarily tuned to allow a little bit of variation to ‘escape’ detection. Is this a hardwired effect to enable pathoadaptation? What would happen to the evolutionary trajectory of PA if this efficiency could be reduced? Just as *mutS* and *mutL* mutants exhibit elevated mutation, what would happen if we could ‘step on the evolutionary brakes’ and induce a state of *hypo* mutation?

vi. Does MutS_PA_/MutL_PA_ oligomerize on the DNA at a mismatch to elicit ‘action at a distance’, as inferred for *E. coli *? [But see also the ‘redox-directed’ solution to the problem outlined in (iii) above.] Is the operation of the MutS_PA_/MutL_PA_ complex the same on both the leading and the lagging strand?

vii. What causes MutS_PA_ to detach from β_PA_ at a mismatch? On a more general level, how does a single protein (MutS) detect the full range of *different* mismatches in DNA? This is significant because there can be up to a ca. 1000-fold differential rate of repair between mismatches, depending on the bases involved;

A:G < C:C < G:A < C:A, A:A, G:G, T:T, T:G, A:C, C:T < G:T, T:C

Increasing sensitivity→

This differential repair rate correlates reasonably well with the affinity of MutS for the different mismatches, although interestingly, deletion of the MMR machinery does not *completely* remove this bias in recognition, but instead, reduces the gap to <4-fold [127].

viii. What orients MutL_PA_ on the heteroduplex? If, as anticipated by analogy with the model developed for *B. subtilis* MMR, MutL activity is integrally-linked with the β-clamp, this may not be easy to establish. Furthermore, how does the UvrD_PA_ helicase (and the subsequent exonuclease activity) know which direction to unwind/digest the DNA?

In summary, and unlike *E. coli *, PA is often associated with long-term (months-years) chronic infection scenarios. In these circumstances, loss-of-function mutations in *mutS*/*mutL* leading to hypermutability are common. However, and in spite of the role(s) played by MMR in PA pathophysiology, the enzymology is far less well-developed (largely for the usual, historical reasons) than it has been in other model organisms such as *E. coli *. This is a shame, because the biochemistry of the MutH-less, methylation-independent PA system seems to us to be far more intriguing. Indeed, a far better comparator may be the Gram-positive organism, *B. subtilis *, whose MMR enzymology appears to more closely approximate to that in PA. As noted above, there are several outstanding research questions that still need to be addressed, and these are not trivial issues either. MMR is far from being a ‘solved problem’, and the next generation of researchers clearly have their work cut out.
